# Acute and Reproductive Effects of Align®, an Insecticide Containing Azadirachtin, on the Grape Berry Moth, *Lobesia botrana*

**DOI:** 10.1673/031.010.3301

**Published:** 2010-04-10

**Authors:** F. Javier Sáenz-De-Cabezón Irigaray, Fernando Moreno-Grijalba, Vicente Marco, Ignacio Pérez-Moreno

**Affiliations:** Unidad de Protección de Cultivos Departamento de Agricultura y Alimentación, Universidad de La Rioja, C/ Madre de Dios, 9 51, 26006-Logroño (La Rioja), Spain

**Keywords:** larval mortality, egg mortality, adult sterility, sublethal doses

## Abstract

Azadirachtin, derived from the neem tree, *Azadirachta indica* A. Juss (Sapindales: Meliaceae), seems promising for use in integrated pest management programs to control a variety of pest species. A commercial formulation of azadirachtin, Align®, has been evaluated against different developmental stages of the European grape berry moth, *Lobesia botrana* Denis and Schiffermüller (Lepidoptera: Tortricidae). When administered orally, Align reduced the fecundity and fertility of adults treated with 1, 5, and 10 mg litre^-1^. At the highest doses, fecundity and fertility were zero, but longevity was not affected. An LC_50_ of 231.5 mg litre^-1^ was obtained when Align was sprayed on eggs less than 1 day old. Hatching of all egg classes was significantly reduced, and this reduction was more pronounced for eggs less than 24 h old. LC_50_ values of 2.1 mg litre^-1^ for first instars and 18.7 mg litre^-1^ for third instars were obtained when Align was present in the diet. Larvae reared on a diet containing different concentrations of Align did not molt into adults at the highest concentrations (0.3, 0.6, 1.2), and 50% molted at the lowest concentration (0.15). Phenotypic effects included inability to molt properly and deformities. The combination of acute toxicity and low, effective concentrations of Align observed in this study could lead to the inclusion of insecticides containing azadirachtin in integrated management programs against this pest.

## Introduction

The grape berry moth *Lobesia botrana* Denis and Schiffermüller (Lepidoptera: Tortricidae), is a major pest of European and Mediterranean vineyards. The yield reductions caused by this insect are due to both damage done by the larvae and further attack by fungi. Most growers control this pest with traditional chemical pesticides, however, mating disruption and microbiological insecticides are used as alternatives in a few areas (Coscollá 1997). Considerable effort is being directed toward reduced use of traditional pesticides and increased use of integrated pest management techniques, emphasizing the joint use of natural enemies and selective pesticides, an alternative compatible with the protection of non-target organisms and the environment. Azadirachtin, a tetranotriterpenoid, is the most active insecticidal compound found in the seeds and leaves of the neem tree, *Azadirachta indica* A. Juss (Sapindales: Meliaceae) (Koul et al. 1990; Schmutterer 1990). This compound exerts several biological effects on insects (e.g. antifeedant, insect growth regulator and repellent) and has low mammalian toxicity (Schmutterer 1990; Ascher 1993; Mordue and Blackwell 1993; Mordue et al. 1998; Mulla and Su 1999; Koul and Wahab 2004; Isman 2006). Pesticides containing azadirachtin are reported to provide broad-spectrum control of more than 200 species of insect pests (Ascher 1993) and to be less toxic to natural enemies of insect pests compared with synthetic, chemical pesticides (Hoelmer et al. 1990; Schmutterer 1997). Although the safety of azadirachtin for numerous beneficial insects has been recently questioned (Spollen and Isman 1996), applications of formulated azadirachtin had no undesired effects on important predators such coccinellids (Banken and Stark 1998), lacewings (Medina et al. 2004) and Phytoseiid mites (Castagnoli et al. 2005; Brito et al. 2006). Thus, pesticides derived from the neem tree seem to be promising for use in integrated pest management programs to control various pest species (Isman 2006).

Grape growers use large amounts of traditional chemical pesticides, and, therefore, the development of strategies that minimize pesticide use is needed. According to the European Directive 98/8/EC, “proper pesticide use includes application at an efficacious concentration and minimization of use of biocidal products where possible.” To ensure sufficient management of pests, registered pesticides are normally applied at rates above the upper asymptote of the concentration/response curve. Thus, in theory, it should be possible to achieve control of insect pests and, at the same time, reduce the effects on non-target insects by using dosage rates below these maxima, with the species-specific degree of selectivity strongly influenced by the applied concentration (Poehling 1989). Published studies of dose reduction from recommended field rates demonstrated adequate levels of control and improved control exerted by natural enemies without sacrificing yield or inflating cost (Smith et al. 1985; Poehling 1989; Acheampong and Stark 2004).

In the only published study of azadirachtin on *L*. *botrana*, Ioriarti et al. (1992) obtained 30% mortality on 3-day-old eggs and 52.6% mortality on larvae, using doses of 500 – 600 mg litre^-1^. Studies are needed to determine the most efficient usage against this pest. Herein the effects of azadirachtin on adult fecundity, fertility and longevity are described. This study also reports the effects of azadirachtin on the development and mortality of *L. botrana* eggs and larvae. Finally, the possibility of incorporating azadirachtin into programs for management of the European grape berry moth are discussed.

## Materials and methods

### Insects

A stock culture of *L. botrana* was established from larvae collected in an organic vineyard in La Rioja, Spain during May 2000, and the culture was augmented with new individuals once each year. The insects were maintained in a growth chamber at 24±1°C, 60±10% RH and 16:8 L:D photoperiod, following the method described by Del Tío (1996) and modified by Sáenz-de-Cabezón (2003). All bioassays were conducted under these uniform conditions.

### Chemicals

Formulated azadirachtin (ALIGN® EC, 32 g. azadirachtin litre^-1^) was obtained in Spain from Sipcam Inagra.

### Effect on adult fecundity, fertility and longevity

To assess the activity of azadirachtin on *L. botrana* adults, three pairs of recently emerged adults were introduced into a 33 ml plastic container placed upside-down on the base of a 90 mm diameter Petri dish. Azadirachtin was administered orally via a water trough that contained 1, 5, and 10 mg litre^-1^ of azadirachtin mixed in a 10% honey solution. Water troughs were changed every 3 days to avoid fungal proliferation. Every day, the glasses with the laid eggs were replaced, and the eggs were then transferred to plastic boxes (12 cm diameter, 5 cm high) until emergence. Fecundity and fertility were counted daily during the oviposition period. Mortality was recorded during the entire life period. Five replicates were used for both the treatment and the control (10% honey solution). Embryonic development was followed using a stereoscopic lupe (Olympus SZH-10).

### Ovicidal bioassays

To test topical ovicidal effects, ten pairs of *L. botrana* adults were introduced into an oviposition chamber consisting of a cylindrical plastic body (9 cm diameter, 13 cm high) covered inside with a transparent plastic film on which females could lay their eggs. The bottom was covered with a Petri dish, and the top was covered with filter paper. Moths were provided with water. Eggs laid during the next 24h were removed and then treated using a manually loaded Potter spray tower, with 5.5 ml in the tank at 20 kPa pressure producing a deposit of 0.05±0.01 ml cm-2. The concentrations that were used were obtained by preliminary assays ranging from 1 to 768 mg litre^-1^. Eggs of different age classes (0–24, 24–48, 48–72 and 72–96 h old) were collected and treated in the same manner. Treatments were made on each of the age groups. The concentration used was the LC_50_ obtained in the previous assay. Egg hatch was recorded 5 days after treatment. There were five replicates per concentration/treatment, in addition to the water treated control.

Ovicidal effects of Align were also examined using a contact bioassay. Surfaces were treated by dipping four plastic glasses in a 1:1 solution of LC_50_ contact concentration, and then air-drying for 5 min. Five pairs of adults were introduced and allowed to lay eggs for 24 h. Larval emergence from eggs was checked at the sixth day of treatment. Four replicates were used for the treatment and the control, which was dipped in distilled water.

### Larvicidal bioassays

To determine the effect of Align on the 1st, 3rd and 5th instars, ten larvae were introduced to a Petri dish (5 cm diameter) containing treated diet at concentrations ranging from 0.75 to 3.8 mg litre^-1^ for 1^st^ instars and 8.0 to 32.0 mg litre^-1^ for 3rd instars. The concentrations used were established by preliminary assays. 5th instars were not treated because of delayed effects in development at ≥ 30 mg litre^-1^. Five replicates were used for each dose and control. The mortality (larvae that failed to molt) was measured when control larvae reached the next stadium (i.e., the 4th and 3rd day after treatment for 1st and 3rd instars, respectively). Sublethal effects of Align exposure were measured during the larval stage by providing 1st instar larvae *ad libitum* diet containing low concentrations of Align (i.e. 0.15, 0.3, 0.6, 1.2 mg litre^-1^). Mortality and adult emergence were checked daily. Five replicates, of 10 individuals each, were used for each concentration and the control.

**Table 1.**
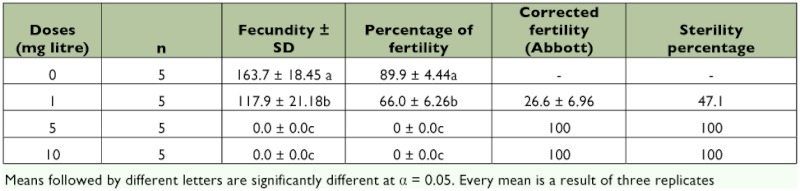
Fecundity and fertility parameters of adult *L*. *botrana* pairs treated *ad libitum* with different concentrations of azadirachtin 1, 5, and 10 mg litre-

### Statistical analysis

Estimates of LC_50_ and LC_90_ for different stages and their 95% fiducial limits were obtained using the POLO program (Russell et al. 1977) based on Finney (1971). The criterion used to estimate the differences between LC ratios was non-overlap of their 95%) confidence intervals (Robertson and Presley 1992). The significance of results (fecundity, fertility, egg mortality, and larval mortality) were tested by ANOVA, and means were separated by an LSD multiple range test (p < 0.05). Percentage values were arcsin transformed, and, in all cases, untransformed means are presented. Abbott's formula was used to correct mortalities (Abbott 1925). Percentage of sterility was calculated using the formula of Toppozada et al. (1966):

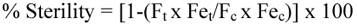

where F_t_ = fecundity of treated females; Fe_t_ = fertility of treated females; F_c_ = control fecundity; and Fe_c_ = control fertility

## Results

### Effects on adults

*L. botrana* adults fed Align in a honey solution showed decreased fecundity and fertility at each concentration tested (Table 1). Total eggs laid per female were significantly (p < 0.05) less than the control, and fertility was significantly (p < 0.05) decreased in *L. botrana* adults. In addition, eggs laid by Align-treated females showed the same toxic symptoms on the embryo as spray-treated eggs (see below). Longevity was not affected by the treatment of any concentration of Align.

### Effects on eggs

The parameters obtained for the probit-log concentration regression line on eggs treated with azadirachtin and less than 24 h are given in Table 2. Contact treatment was significantly (p < 0.05) more effective than surface treatment (with 41.4 ± 4.3 and 30.0 ± 7.1 corrected mortalities, respectively). Treatment with Align significantly (p < 0.05) reduced hatching for all egg classes but the last (72–96 h old). The reduction was greater for eggs less than 24 h old than for other egg classes (Table 3). High concentrations of Align (768 mg litre^-1^) interrupted the normal development of the embryo. Treated eggs reached the final black-head stage but had mandibular and cephalic deformities.

**Table 2.**
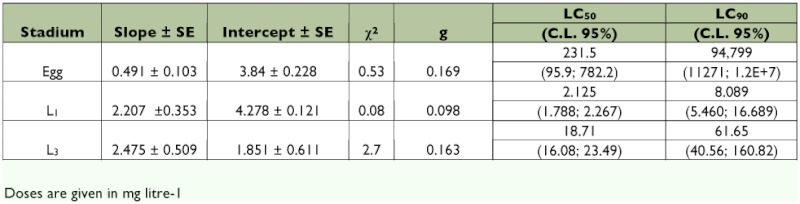
Probit-log concentration-response regression line parameters, of different stages of *L*. *botrana* treated with azadirachtin.

### Effects on larvae

Table 2 shows probit-log concentration regression line parameters obtained for the different stages tested. Repellent effects of Align were evident during the course of preliminary assays, when death due to starvation was evident in feeding experiments with concentrations higher than 12 mg litre^-1^. Younger larvae were more susceptible than older larvae. Effects on larval development were observed and included the inability to shed the old cuticle and cephalic capsule. In addition, larvae that successfully molted exhibited mouthpart deformities, integument injuries and malformations. Treatment of 5th instars with concentrations higher than 32 mg litre^-1^ of Align caused larvae to prematurely enter a wandering state and molt the pupal stage. Hardening the cuticle sclerites and other abnormalities, such as larval-pupal intermediates, were also observed. When treated with reduced concentrations of Align, there was a high percentage of larval mortality in the 5th stadium for all concentrations tested except 0.15 mg litre^-1^. Mortality on the next stadia reached >85% in all concentrations but 0.15 mg litre^-1^. Almost 100% of the individuals died without reaching adulthood at concentrations above 0.15 mg litre^-1^. The lowest concentration reduced adult elosion up to 50%. Mortality occurred mostly during the last larval stage (Figure 1).

## Discussion

Align®, a commercial formulation of azadirachtin, has larvicidal and ovicidal activity and significantly affected the reproduction of *L. botrana.* At low concentrations total adult eclosion was reduced to 0%.

**Table 3.**
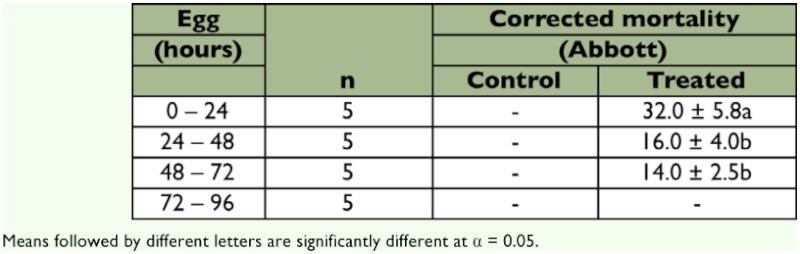
Hatching percentage and corrected egg mortality of four age classes of *L*. *botrana* treated with 231.5 mg litre of azadirachtin.

**Figure I.**
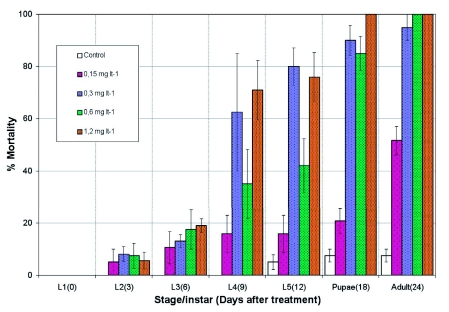
Stage-instar mortality when 1st instar larvae of *Lobesia botrana* are treated *ad libitum* during the larval stage with low concentrations of azadirachtin (mg litre). High quality figures are available online.

In a previous study with this insect, Iorriati et al. (1992) measured mortalities of 30% for 3-day-old eggs and 52.6% for larvae using concentrations between 500 and 600 mg litre^-1^. In the present study the effects of Align on eggs, larvae and adult *L. botrana* have been assessed, emphasizing the time and mode of treatment.

### Effects on adult fertility, fecundity, and longevity

Align strongly affected the reproduction of *L*. *botrana* by producing a high percentage of sterility at the concentrations tested. Fecundity was severely reduced, and no eggs were laid at the highest concentrations tested. Lower concentrations significantly reduced the fertility of *L. botrana.* Eggs laid by treated females showed embriocidal effects, which suggest pesticide transference through the gravid female. It has been demonstrated that adult exposure to azadirachtin by topical, surface or ingestion treatments, reduces fecundity and/or fertility among different insect orders, including Lepidoptera (Schmutterer 1990; Ascher 1993; Mordue and Blackwell 1993; Mordue 2004; Seljansen and Meadow 2006). It is known that azadirachtin causes profound effects on reproductive processes of both male and female insects. Studies showed that insects treated with azadirachtin have degenerate ovaries and a high degree of yolk resorption (Rembold and Seiber 1981; Koul 1984; Dorn et al. 1987; Schlüter 1987; Medina et al. 2004). Azadirachtin also interferes with the synthesis of vitellogenin by the fat body and its uptake by the eggs, resulting in reduced fecundity and sterility. Such effects could be due to the disruption of juvenile hormone levels and ovarian ecdysteroid production (Rembold and Seiber 1981; Feder et al. 1988; Tanzubil and McCaffery 1990).

### Ovicidal effects

Align was not a potent ovicide of *L. botrana* eggs (LC_50_ of 231.5 mg litre^-1^ for eggs < 24 h). Ovicidal activity was also affected by age and treatment. Younger eggs were more susceptible than older ones, and surface treatment was less effective than spray treatment. These findings are in agreement with those from previous studies showing that azadirachtin has low ovicidal activity on *L. botrana* (Iorriati et al. 1992) and on insects of different orders (Schmutterer 1990; Ascher 1993). The results from the present study differ from previous studies (Ioriartti et al. 1992) that obtained a greater susceptibility on 3-day-old eggs.

### Larvicidal effects

*L. botrana* larvae were highly susceptible to diet containing Align, with younger stages being more susceptible than older ones. These results agree with those from previous studies (Mordue and Blackwell 1993) of other lepidopteran insects showing larvicidal activity inversely proportional to larval age. In addition to increased mortality, Align acted as an insect growth regulator and an antifeedant: 1st instars did not fed when concentrations were higher than 12 mg litre^-1^ (data not shown) (Scmutterer 1990; Ascher 1993; Mordue and Blackwell 1993). Also, 5th instars displayed premature wandering and cessation of feeding, possibly due to Align's effects on release of prothoracicotropic hormone. As described for other species, an increase in the levels of prothoracicotropic hormone is necessary to begin the transformation into pupa. Without this increase, the larvae remain in a state of wandering and die (Schmutterer 1990; Mordue 2004). In a study on *Tripanosoma cruzi*, the complete elimination of azadirachtin did not occur until 50 days after the treatment. Thus, this compound can interrupt the insect development for a long time (González and Garcia 1992). Low concentrations of Align resulted in elevated mortality during the last larval stage, probably as a result of compound accumulation reaching concentrations that have activity in older instars.

### Integrated Pest Management

The main impediment for the implementation of integrated pest management is to provide growers with a convincing reason to adopt its tactics, such as economic incentives (Reitz et al. 1999). Integrated pest management often increases net profits for growers who adopt it; however, many growers still hold the perception that it does not offer short-term economic advantages compared with conventional control. However, in a previous study (Sáenz-de-Cabezón 2003), Align was as effective as the conventional organophosphate fenitrothion, even when applied at a concentration (50 cc/hl) lower than that recommended on the label (125 cc/hl; data not shown). Thus, proper pesticide-use tactics reduce the cost of pesticide use and further the implementation of integrated pest management programs.

More laboratory, small plot and field testing is required before the insecticidal potential of azadarachtin against *L. botrana* will be fully understood. The activity observed in this study suggests that the combination of the acutely toxic and reduced concentrations could lead to the incorporation of this compound in integrated pest management programs against the grape berry moth. Sprays for the second and third generations should be applied at the beginning of flight, to cover the oviposition period and egg hatching. Resistance studies are needed in order to know how azadarachtin resistance works in *L. botrana* populations using different levels of selection pressure.
